# Real-Time Foot Tracking and Gait Evaluation with Geometric Modeling

**DOI:** 10.3390/s22041661

**Published:** 2022-02-20

**Authors:** Ming Jeat Foo, Jen-Shuan Chang, Wei Tech Ang

**Affiliations:** 1 Rehabilitation Research Institute of Singapore, Singapore 308232, Singapore; jenchangnus@gmail.com (J.-S.C.); wtang@ntu.edu.sg (W.T.A.); 2 School of Mechanical and Aerospace Engineering, Nanyang Technological University, Singapore 639798, Singapore

**Keywords:** gait evaluation, human motion, modeling, object tracking, RGB-D camera, depth camera

## Abstract

Gait evaluation is important in gait rehabilitation and assistance to monitor patient’s balance status and assess recovery performance. Recent technologies leverage on vision-based systems with high portability and low operational complexity. In this paper, we propose a new vision-based foot tracking algorithm specially catering to overground gait assistive devices, which often have limited view of the users. The algorithm models the foot and the shank of the user using simple geometry. Through cost optimization, it then aligns the models to the point cloud, showing the back view of the user’s lower limbs. The system outputs the poses of the feet, which are used to compute the spatial-temporal gait parameters. Seven healthy young subjects are recruited to perform overground and treadmill walking trials. The results of the algorithm are compared with the motion capture system and a third-party gait analysis software. The algorithm has a fitting rotational and translational errors of less than 20 degrees and 33 mm, respectively, for 0.4 m/s walking speed. The gait detection F1 score achieves more than 96.8%. The step length and step width errors are around 35 mm, while the cycle time error is less than 38 ms. The proposed algorithm provides a fast, contactless, portable, and cost-effective gait evaluation method without requiring the user to wear any customized footwear.

## 1. Introduction

Mobility impairment is one of the most prominent health problems among senior citizens in Europe and the USA [1]. Gait parameters, such as step length and step width, can be used to evaluate the recovery performance of patients [2,3]. Moreover, the Base of Support (BoS), constructed from the relative positions of the feet, is often used with the eXtrapolated Center of Mass (XCoM) to evaluate balance [4,5]. Thus, gait analysis is important in gait rehabilitation and assistance for a more thorough and comprehensive assessment. These measurements require the precise localization of the feet in 3D space, which is difficult to perform in an unstructured environment [6,7,8].

Currently, there are many approaches for gait analysis. The gold standard is the marker-based motion capture system, which is costly and requires a laboratory setting. Other solutions include the floor sensor approach that utilizes pressure sensors inside an instrumented walkway; the gait is measured through the ground reaction forces when the subject walks on it [9,10]. This approach is not restricted to a laboratory environment, however the workspace is still limited to the length of the walkway.

Wearable gait analysis tools provide an infinite workspace for gait assessment and reduce operational complexity. Some examples are special shoes equipped with force sensors and the implementation of inertial measurement units [11]. Nonetheless, they require the sensors to be attached to the user, which have low usability and may be uncomfortable. Hence, portable, low-cost, and contactless sensors have been developed to solve the problem. The feasibility of Red Green Blue Depth (RGB-D) cameras in contactless foot tracking and gait evaluation is commonly studied nowadays. While many were developed for general clinical applications [12,13,14,15,16], few literature targets the field of assistive technologies, which have limited view of the subjects.

Some researchers have used a Kinect camera to view the lower limbs of a walker user. Hu et al. adopted a model composed of tapered cylinders to model the thigh and calf, and half-cylinders to model the feet [17]. They employed a probabilistic approach based on particle filtering for the tracking and achieved less than 4 mm of step length error with 14–19 s of processing time per frame. Joly and their team modeled the leg with a cylinder and the foot as a plane, connecting each other with a ball joint [18]. The feet and the legs were segmented from the background to fit into their respective models using optimization. The elevation and bearing angle were then computed. The elevation angle had less than a $5^{\circ }$ error in most cases while the bearing angle was less accurate. Zhang and Ye modeled the legs with a skeletal system [19]. The lower limbs were segmented using region growing and the results were fitted to multiple least square planes. The authors then used the Markov Chain model and Support Vector Machine (SVM) to identify walking patterns. Without the use of modeling, the feet can still be directly extracted from the depth image [20,21]. The position of the foot was determined to be its centroid, whereas the orientation was found using Principal Component Analysis (PCA). The position error along the anterior-posterior direction and the orientation error with respect to the vertical axis were less than 31 mm and 22 Root Mean Square Deviation %, respectively for healthy subjects [20] and 40.1 mm for the position error along the forward for older adults [21].

Notwithstanding, all the mentioned applications view the feet from the front and above, which may be appropriate for robots that position in front of the user, however are not suitable for devices like over-ground gait rehabilitation robots or assistive devices that follow the user from the back [22,23,24,25]; the methods may not be easily transferable since the forefeet are generally not visible to the camera for the case of such devices.

A deep neural network, termed SDF-Net was proposed in [26] and was integrated into a foot tracking algorithm as a preliminary version of this work [27]. To the best of authors’ knowledge, this work is the first to track the feet when viewing the lower limbs from the back. However, the results were limited to only a single subject and on a particular shoe model. The work also did not publish any real gait results.

In this paper, the authors propose a new algorithm utilizing geometric models to track the feet from the back view of the lower limbs point cloud. The shank and foot of the subject are modeled using simple geometry. The algorithm is designed to track a subject’s feet without having to prepare the necessary neural network prior to the application. Instead, it provides a more generic way to track the feet of different sizes and shapes. The algorithm aligns the 3D models to the point cloud of the tracked subject through optimization. The subjects’ gait parameters are then extracted from the tracking results. The method requires only a CPU to run, making it easier to be implemented on various rehabilitation and assistive devices. The performance of the algorithm is evaluated with a motion capture system and a third-party gait evaluation software.

## 2. Materials and Methods

This section first describes the foot tracking algorithm, presenting the procedures of model construction, object recognition, and cost optimization for object localization. From the results of the tracker, the spatial-temporal gait parameters are calculated to evaluate the walking performance.

### 2.1. Foot Tracking

**Figure 1:**
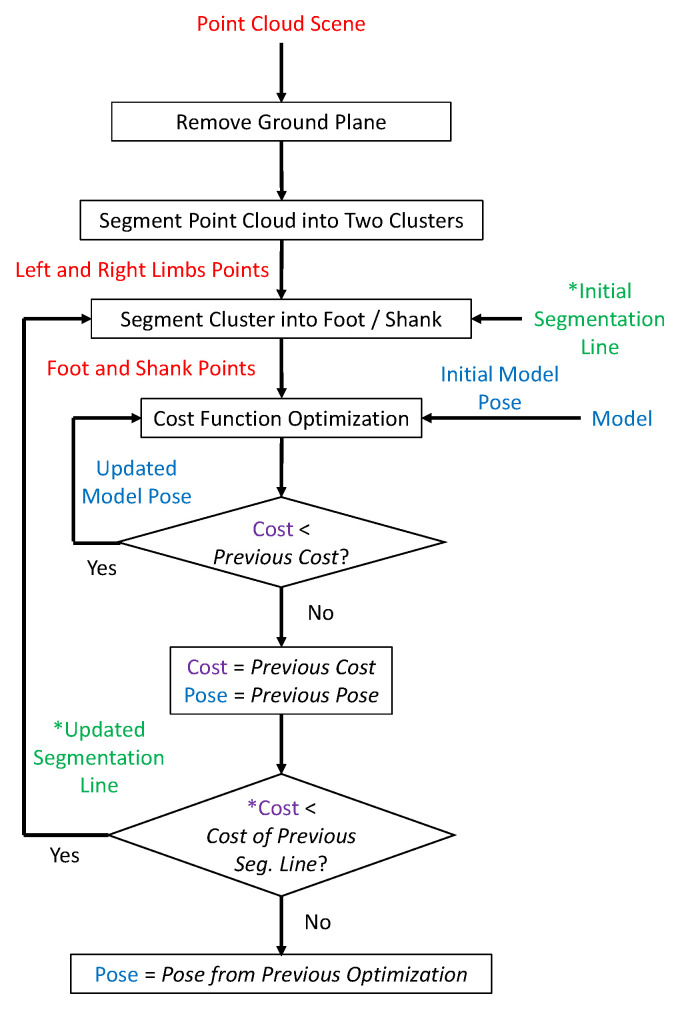
The flowchart of the foot tracking process. The blocks with an asterisk (*) are only applicable to expansion-segmentation, i.e., these process are not applicable to contour-segmentation.

Figure 1 illustrates each step in the foot tracking process.

#### 2.1.1. Model Construction

**Figure 2:**
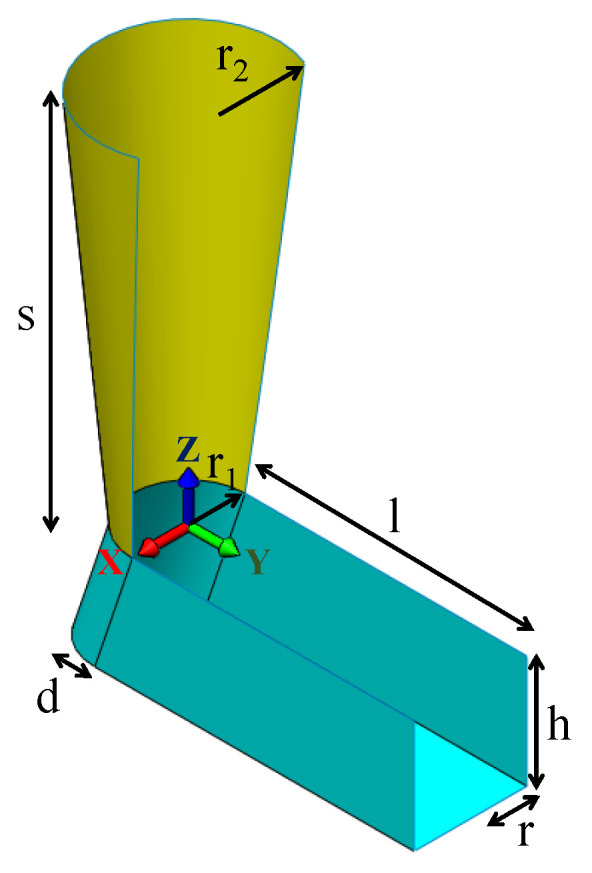
The foot-shank model used in the geometric model-based foot tracker. The foot and shank are colored cyan and yellow, respectively. The foot model is parameterized by four parameters, namely the radius of the heel *r*, the height of the foot *h*, the foot length *l*, and an offset *d* at the heel which models the calcaneus bone. The shank model is parameterized by the lower shank radius $r_{1}$, the upper shank radius $r_{2}$, and the shank length *s*. The origin of the models is located at the center of the intersection plane between the shank and foot. The two models share the same position but can have different orientations.

The proposed model is designed to model the foot and the shank when viewing the subject from the back (Figure 2). The foot is modeled as a U-shape prism with the top and front faces removed as they do not conform to the actual foot shape. The U-shaped portion fits the heel of the foot and is parameterized by radius *r*. The foot length and height are controlled by two parameters, *l* and *h*, respectively. As there is a protruding feature at the heel due to the calcaneus bone, a protrusion offset *d* is added.

From our preliminary tests, tracking the feet using only the foot model often yields erroneous results as the feet only occupy a small portion of the image, causing the model to easily fit to the other parts of the image. Moreover, it is difficult to relocate the foot without human intervention if the algorithm loses track. The system then utilizes the easily recognizable cylindrical feature of the shank to guide the tracker. The shank is modeled as a tapered-cylinder parameterized by two radii $r_{1}$ and $r_{2}$, and the length *s*. Since only the back of the shank can be seen by the camera, the front part of the cylindrical model is removed to prevent incorrect fitting.

The top part of the foot model and the bottom part of the shank model are joined; the two models share the same position in the 3D space. However, they are free to rotate with respect to each other.

#### 2.1.2. Object Recognition

To perform the tracking, the pixels that are related to the objects of interest, i.e., the shank and the foot, must be identified. The procedure begins with the pre-processing of the depth image. Firstly, the point cloud of the scene is reconstructed from the depth image. Points that are more than 1.0 m away from the camera are removed. The ground plane is then identified so that only points above the ground plane are considered in the next step.

**Figure 3:**
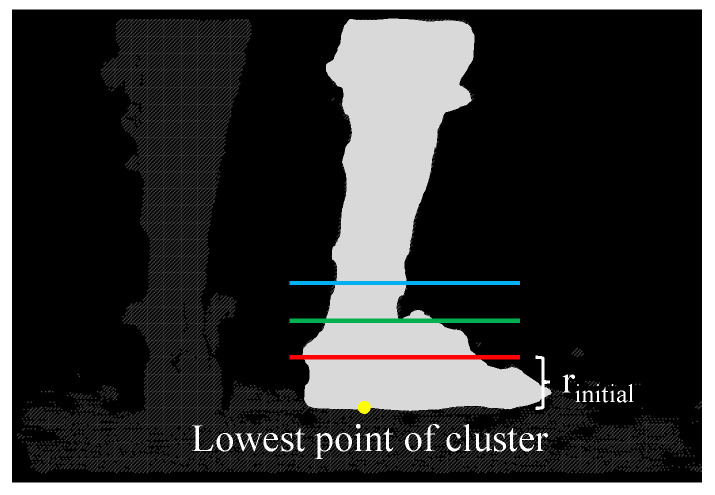
The figure shows a typical scene of the application in the point cloud space. The expansion-segmentation algorithm intends to find a line that separates the right foot from the shank. The lowest point of the right leg cluster is first located (marked yellow in the diagram). In the first iteration, the Foot-Shank Separation Line is assumed to be $r_{initial}$ rows above the point (indicated by the red line). All cluster points below the line are labeled as the foot, while those above are labeled as the shank. The points are then input into the algorithm to obtain a fitting cost. The algorithm now enters an iterative process in which a new line above the previous line is selected as the new Foot-Shank Separation Line (indicated by the green line) and a new cost is computed. The iteration stops when the cost is no longer decreasing with another new line (indicated by the blue line) and hence the previous line is determined as the actual separation that differentiates the foot from the shank.

Connected points with similar depth are formed into clusters. It is assumed that the lower limbs contribute to the two largest objects in the image and there are no other large objects present in the scene. Hence, the clusters are sorted into two groups using the K-means algorithm. The left and right limbs are identified according to the positions of the two groups in the image. At this stage, the points corresponding to the foot or the shank, however, are yet to be identified. The foot -shank segmentation is achieved by implementing a region growing mechanism inspired by [18]. It is assumed that the two clusters consist entirely of the lower limbs, which include the feet and the shanks of the person. The mechanism aims to identify the body part each pixel belongs to. To begin with, the depth image is examined. A line which is $r_{initial}$ rows above the lowest point of a group is marked as the Foot-Shank Separation Line, such that all points below the line are assigned to the foot model, whereas the points above the line are assigned to the shank model. These points are then loaded into the optimization algorithm (to be presented later) to localize the foot and the shank; the optimization function outputs a cost indicating the fitting quality. In each of the subsequent iterations, the Foot-Shank Separation Line is moved upward. The foot and shank pixels are labeled similarly, and a new cost is computed. The process is repeated until the output cost does not decrease further, indicating that the actual Foot-Shank Segmentation Line has already been found. The algorithm, termed as expansion-segmentation, is illustrated in Figure 3.

**Figure 4:**
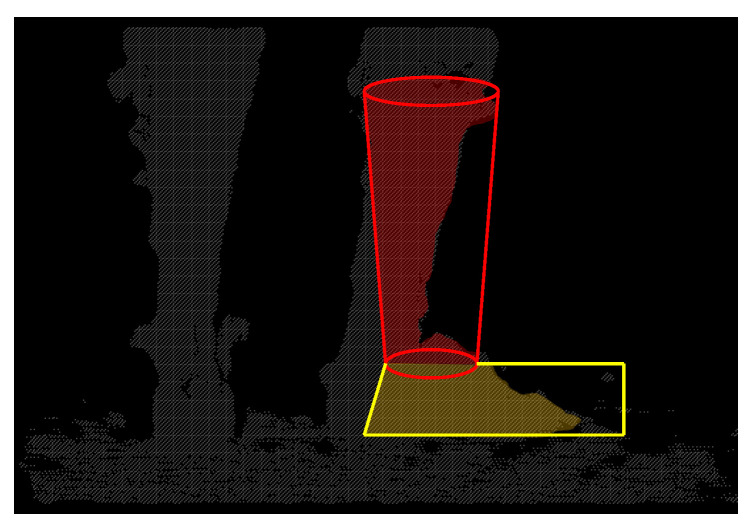
The figure illustrates the depth pixels that are sampled in contour-segmentation (shaded red and yellow). The geometric models are projected onto the depth image plane to form the 2D outlines of the models. The pixels within the outlines are examined. If the corresponding 3D points lie near to the surface of the respective models, the pixels are labeled accordingly.

Nonetheless, there are scenarios in which only one cluster is found, which causes the expansion-segmentation to fail. This is generally caused by two reasons: (i) One of the lower limbs is occluded by the other, which occurs when the person is turning or walking too close to the camera, or (ii) the two legs come into contact with each other, causing the algorithm to identify them as one big cluster. Hence, another object identification method, which is termed as contour-segmentation, is used to label the pixels under these scenarios. The vertices of the foot and shank models are projected onto the 2D image plane. Two convex hulls are formed from the respective projected points. The pixels inside the hulls are labeled as foot or shank pixels if their corresponding 3D points lie near to the surface of the respective models (Figure 4).

#### 2.1.3. Cost Optimization

After the data points are sorted into the foot and shank, the poses of the lower limbs with respect to the camera are to be found. The sorted data points are input into their respective optimizers, which find the optimal pose parameters that will produce the lowest cost function.

**Figure 5:**
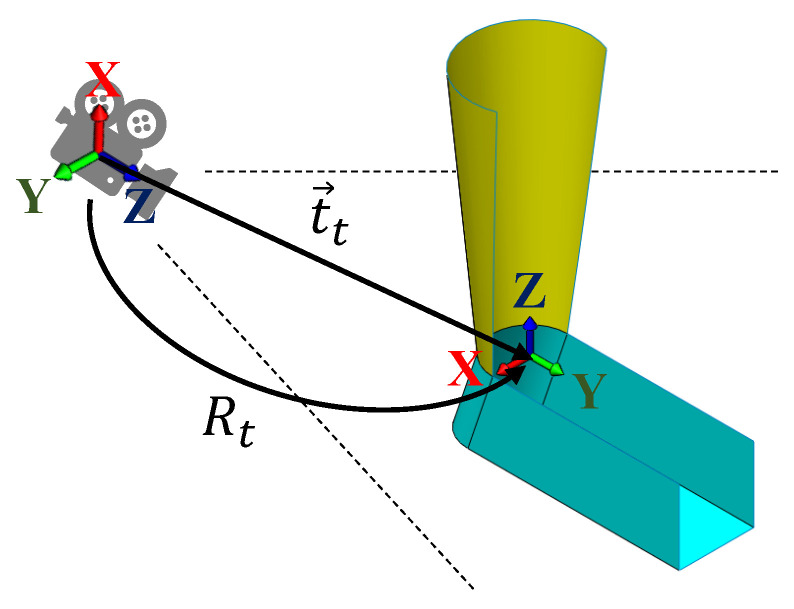
The poses of the models with respect to the depth camera.

For computational efficiency, instead of directly updating the pose parameters, the algorithm updates the change of the pose parameters instead. The finalized poses output by the algorithm in frame *t* are defined as a three-dimensional rotation matrix, $R_{t}$, and a three-dimensional vector, ${\vec{t}}_{t}$, as illustrated in Figure 5.

In the first iteration of frame *t*, the current rotation and translation ${\tilde{R}}_{t}$ and ${\tilde{\vec{t}}}_{t}$ are initialized to be $R_{t-1}$ and ${\vec{t}}_{t-1}$, respectively. Then in the subsequent iterations, the optimizer updates the changes ΔR and $\Delta \vec{t}$, which are then integrated into the current pose parameters:

(1) $${\tilde{R}}_{t,i+1}={\tilde{R}}_{t,i}\Delta R^{⊤},\;\;\;\;\;{\tilde{\vec{t}}}_{t,i+1}={\tilde{\vec{t}}}_{t,i}+\Delta \vec{t}$$

where *i* is the iteration count, which is omitted in the subsequent text. The changes in pose parameters are then reset to the identity rotation and the zero vector for the next iteration.

Model-based tracking is often seen as an alignment optimization, where the model is aligned to the observation by minimizing their discrepancy [28]. Nonetheless, relying solely on the alignment usually causes the model to be trapped in local minima. Hence, some auxiliary costs are added to facilitate the tracking process, as well as to ensure the solutions to be realistic.

Therefore, the final cost function *E* is defined by three components:

(2) $$E=E_{foot}+E_{shank}+E_{additional}$$

which are the respective fitting costs from the foot and shank models, as well as some additional costs which help to produce more realistic outputs. This subsection describes every term employed in the respective fitting costs.

##### Foot Fitting Cost

The pose parameters of the foot at frame *t* are defined as a rotation matrix $R_{foot,t}$ for orientation and a three-dimensional vector ${\vec{t}}_{model,t}$ for translation. However, for ease of computation, the rotation is transformed into a quaternion ${\vec{q}}_{foot,t}$. In the camera frame, *C*, the observed data points p→tC, are transformed into the model frame, *M*, as p→tM in all computations below unless stated otherwise:

(3) p→tM=ΔRfoot,tR˜foot,t⊤p→tC−(t→˜model,t+Δt→model,t).

The subscript *t* is dropped in the following text to avoid clutter.

The fitting cost of the foot model is given to be:

(4) $$\begin{array}{c} E_{foot}=E_{dist,foot}+w_{1}E_{silhouette,foot}+w_{2}E_{ground} \end{array}$$

where the *w* terms represent the weights of the respective costs.

Before computation, the positions of the observed points with respect to the current model are identified. As the model is constructed geometrically, the closed-form point-to-model distances can be calculated. The term $E_{dist,foot}$, which is the sum of the distances of each sampled point to the foot model can be computed as follows:

(5) $$E_{dist,foot}=\sum_{\vec{p}\in P_{foot}}^{}D\left(\vec{p}\right)$$

where $P_{foot}$ is the set that contains the sampled foot points that are transformed into frame *M*, and D(·) represents the point-to-model distance for that particular point.

The silhouette term $E_{silhouette,foot}$ penalizes the model when it does not fall within the silhouette of the observation points when projected onto the 2D image plane.

(6) $$E_{silhouette,foot}=\sum_{\vec{u}\in X_{FM}}^{}\min_{\vec{x}\in X_{foot}}{∥\vec{u}-\vec{x}∥}_{2}$$

where $X_{FM}$ is a set in $R^{2}$ containing the key points of the foot model, that are transformed into frame *C* and projected onto the image plane, $X_{foot}$ is the set of sampled foot pixels, and ${∥\cdot ∥}_{2}$ denotes the $l_{2}$ norm. This term prevents the model from moving out of the observation point cloud, especially when the feet are facing straight forward and hence poses ambiguity in the orientation.

During the fitting, the front of the foot model is often found to be dipping into the ground when only one side of the foot is seen by the camera. To have a physically realistic pose that does not impose collision to other objects, $E_{ground}$ is imposed to penalize the penetration of the model into the ground plane as:

(7) $$\begin{array}{c} E_{ground}=\sum_{\vec{u}\in P_{FM}\cap P_{below}}^{}N\left({\tilde{R}}_{foot}\Delta R_{foot}^{⊤}\vec{u}+\left({\tilde{\vec{t}}}_{model}+\Delta {\vec{t}}_{model}\right),{\vec{n}}_{ground},{\vec{c}}_{ground}\right) \end{array}$$

where $P_{FM}$ is the set of the key points of the foot model, $P_{below}$ is the set of points below the ground plane, ${\vec{n}}_{ground}$ is the normal of the ground plane, ${\vec{c}}_{ground}$ is a point on the ground plane, and $N(\vec{p},\vec{n},\vec{c})$ represents the point-to-plane distance.

##### Shank Fitting Cost

The orientation of the shank is represented with Euler angles such that the rotation matrix is given to be:

(8) $$R_{shank}=R_{x}R_{y}R_{z}$$

with $R_{x}$, $R_{y}$, and $R_{z}$ as the basic rotation matrices. As only the points on the cylinder with negative *y* normals can be seen by the camera, the rotation about the z-axis is dropped from the calculation such that the orientation is only determined by the angles ${\vec{\theta }}_{shank}=\left\{\theta_{x},\theta_{y}\right\}$; the shank model shares the same translation vector ${\vec{t}}_{model}$ with the foot model.

The fitting cost of the shank model is given to be:

(9) $$\begin{array}{c} E_{shank}=E_{dist,shank}+w_{3}E_{silhouette,shank}+w_{4}E_{orientation}. \end{array}$$

The terms $E_{dist,shank}$ and $E_{silhouette,shank}$ are defined similarly to their foot counterparts:

(10) $$E_{dist,shank}=\sum_{\vec{p}\in P_{shank}}^{}D\left(\vec{p}\right)$$

(11) $$E_{silhouette,shank}=\sum_{\vec{u}\in X_{SM}}^{}\min_{\vec{x}\in X_{shank}}{∥\vec{u}-\vec{x}∥}_{2}$$

where $P_{shank}$ is the set that contains the sampled shank points that are transformed into frame *M*, $X_{SM}$ is a set in $R^{2}$ containing the key points of the shank model, that are transformed into frame *C* and projected onto the image plane, and $X_{shank}$ is the set of sampled shank pixels.

During walking, the tilting of the leg oscillates on the sagittal plane. This sometimes causes the model to be trapped in a local minimum where the model is unable to “straighten” anymore. Adding an orientation term $E_{orientation}$ helps to keep the shank model upright while not affecting the tilting of the shank model significantly. $E_{orientation}$ penalizes the cost function when the leg is not pointing along the vertical direction:

(12) $$E_{orientation}=1-\left({\tilde{R}}_{shank}\Delta R_{shank}^{⊤}*{\left(0,0,1\right)}^{⊤}\right)\cdot {\vec{n}}_{ground}.$$

##### Additional Fitting Cost

Additional constraints are imposed to obtain more realistic optimization outcomes, which are defined as follows:

(13) $$\begin{array}{c} E_{additional}=w_{5}E_{limit}+w_{6}E_{temporal,rot}+w_{7}E_{temporal,trans}. \end{array}$$

In the common scenario, the leg should be standing straight such that the shank orients vertically while the foot is pointing forward and flat to the ground, as illustrated in Figure 2. While the ankle joint allows dorsiflexion and plantarflexion, as well as inversion and eversion, it was found that imposing a term $E_{limit}$ that penalizes these movements helps to stabilize the fitting process. $E_{limit}$ is hence defined to be:

(14) $$\begin{array}{c} E_{limit}=\left({\tilde{R}}_{shank}\Delta R_{shank}^{⊤}{\left(0,0,1\right)}^{⊤}\right)\cdot \left({\tilde{R}}_{foot}\Delta R_{foot}^{⊤}{\left(0,1,0\right)}^{⊤}\right)\\ \;\;\;\;\;\;\;\;\;\;\;\;+\left({\tilde{R}}_{shank}\Delta R_{shank}^{⊤}{\left(0,0,1\right)}^{⊤}\right)\cdot \left({\tilde{R}}_{foot}\Delta R_{foot}^{⊤}{\left(1,0,0\right)}^{⊤}\right) \end{array}$$

where the first and second terms measure the dorsiflexion-plantarflexion and inversion-eversion of the foot, respectively.

As human motion is slow during normal walking, the change of pose between frames will not be significant. Hence, to prevent the algorithm from outputting a pose which is very different from the previous one, a temporal penalty is enforced as follows:

(15) $$\begin{array}{c} E_{temporal,rot}=Q\left(R_{foot,t-1},{\tilde{R}}_{foot}\Delta R_{foot}^{⊤}\right)+Q\left(R_{shank,t-1},{\tilde{R}}_{shank}\Delta R_{shank}^{⊤}\right) \end{array}$$

(16) $$E_{temporal,trans}={∥{\vec{t}}_{model,t-1}-\left({\tilde{\vec{t}}}_{model,t}+\Delta {\vec{t}}_{model,t}\right)∥}_{2}$$

where Q(·) finds the smallest angular distance between two rotations.

##### Optimization Method

The cost function is optimized using the Levenberg–Marquardt (LM) algorithm.

The optimization process is iterated until the cost function (Equation (2)), its change, or the magnitude of parameter changes falls below their respective thresholds, or the number of iterations has reached the maximum number of iterations, $n_{max}$.

To smoothen the trajectory and reject outliers, a Kalman filter is implemented for the pose parameters.

### 2.2. Gait Parameters Computation

Human’s gait cycle consists of two phases, namely the stance phase and swing phase. The gait cycle starts with the stance phase when the foot first contacts the ground, termed the heel-strike (HS). The stance phase ends when the foot leaves the ground at toe-off (TO), beginning the swing phase. When the swinging foot lands the ground, the current gait cycle terminates and another gait cycle begins. From these gait events, gait parameters can be derived. Step length and step width are measured during the double stance phase, in which both feet are on the ground. Step length is defined as the distance between the two feet along the moving direction while step width is defined as the distance between the two feet along the direction perpendicular to the moving direction. Cycle time indicates the duration of the gait cycle; stance time and swing time represent the duration of the stance and swing phase, respectively. The next step of the experiment is to investigate if the algorithm can identify gait events/phases and extract gait parameters accurately.

In the application, the swing and stance phases are determined from the output foot poses. An intuitive approach is to use the distance between the foot model and the ground plane to determine gait events. However, the method is prone to false alarms and misdetection when the fitting is not stable, especially when the foot is far from the camera or when it is obstructed by the other foot. The anterior-posterior (AP) distance of the foot from the camera was found to be a more reliable parameter. When the foot is the furthest away from the camera, it experiences a HS whereas TO occurs when the foot is closest to the camera before swinging forward. These two events are determined from the local maxima and minima of the AP distance of the foot.

In the study, the detection window is set to be 19 depth camera frames, corresponding to an approximate latency of 0.2 s. To minimize the effect of noise, the frame difference between the local minima and maxima has to be more than $\Delta d_{min}$ frames for it to be considered as valid.

Each HS event marks the end of a gait cycle and the start of the other; the gait parameters for that gait are then computed. In this experiment, the actual walking direction ${\vec{v}}_{dir}$ is assumed to be straight forward. Let the displacement vector between the feet be ${\vec{d}}_{step}$, the spatial gait parameters can be computed as follows:

(17) $$d_{steplength}=\left|{\vec{d}}_{step}\cdot {\vec{v}}_{dir}\right|$$

(18) $$d_{stepwidth}=\sqrt{{∥{\vec{d}}_{step}∥}^{2}-d_{steplength}^{2}}.$$

From the timings of HS and TO, the cycle time, the stance time, and the swing time can be computed.

### 2.3. Experiment Protocol

In the experiment, seven healthy young subjects without known lower limb pathology or balance impairment (age: 27.5±3.1, height: 171.2±6.9 cm, weight: 68.1±4.5 kg, 1 female) were recruited to perform static and walking trials, yielding nearly 50,000 frames for fitting and more than 1200 steps. The walking trials were done in two conditions, the first one being treadmill walking. The gait speed of healthy population aged 60–99 ranges from 0.74 m/s to 1.18 m/s [29,30]. For pathological gaits, chronic stroke patients normally walk at speeds between 0.4 and 0.6 m/s [31]. Studies have shown that patients who walk slower than 0.6 m/s require the help of caregivers in performing activities of daily living [32], whereas individuals who walk at 0.2–0.4 m/s require assistance entering and exiting structures and have difficulty in stairs climbing [31]. Hence, two treadmill speeds of 1.0 m/s and 0.4 m/s were tested to simulate the gait patterns of the target population. The second condition involved overground walking with an overground gait assistive robot, Mobile Robotic Balance Assistant (MRBA) [25], which serves as a test case for assistive device implementation. In this condition, the subjects walked at their preferred speed. MRBA is connected to the pelvis of the subject during used. It is designed to follow the subject while helping them to maintain their balance when they perform activities of daily living.

The recorded actions are summarized as follows:

1. A static trial in which the subject stands straight with feet pointing forward for at least 5 s;
2. Treadmill walking at normal speed (1.0 m/s) (T10) for 30 s for three trials;
3. Treadmill walking at low speed (0.4 m/s) (T04) for 30 s for three trials;
4. Overground Walking (OW) consisting of a 7 m straight walking path with MRBA for six trials.

**Figure 6:**
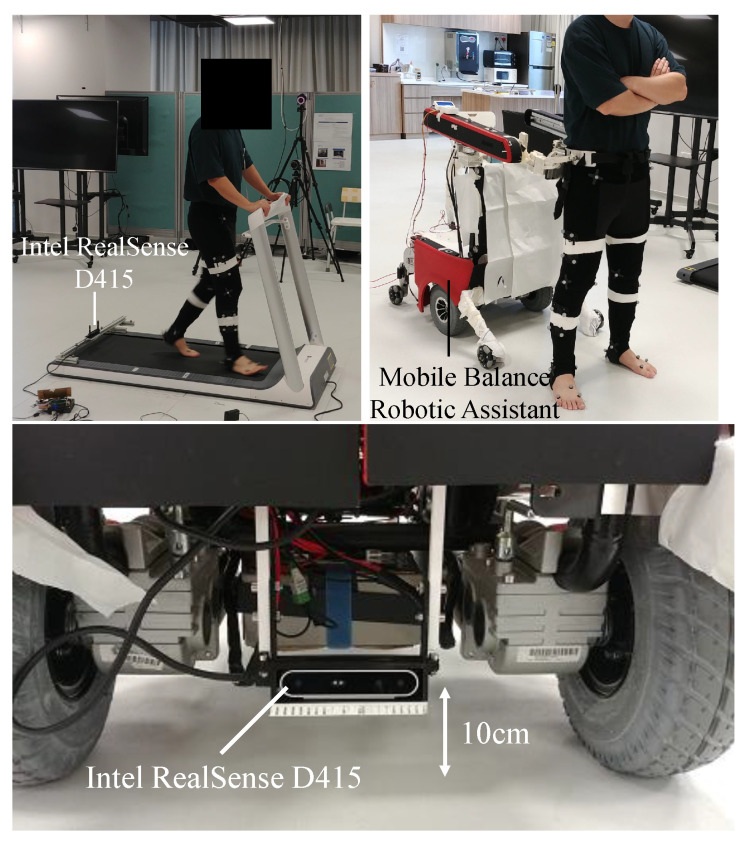
**Top Left:** The setup of treadmill trial. **Top Right:** The setup of overground trial with an overground gait assistive robot, Mobile Robotic Balance Assistant (MRBA) [25]. **Bottom** The mounting of the RGB-D camera Intel RealSense D415 on MRBA.

Throughout the experiment, an RGB-D camera Intel RealSense D415, installed 10 cm above the walking surface, was used to capture the recording of the feet from the back at 50 fps; the camera was integrated into MRBA for the case of overground walking. Prior to the overground walking trial, a 3-min practice session was allowed for the subject to learn how to interact with the robot. The experiment setup is shown in Figure 6.

The shapes of the feet and shank of the subjects were measured according to the parameters shown in Figure 2 to construct the lower limb model.

To compare the results of the proposed algorithm with the motion capture system (Qualisys Miqus M3 (2MP)) [33], infrared (IR) markers were placed on the lower body of the subjects according to the CAST lower body marker set [34]. The proposed algorithm does not require any marker placement.

All subjects were required to sign a consent form approved by the Institutional Review Board, Nanyang Technological University, Singapore (Application ID: IRB-2019-06-016).

### 2.4. Data Analysis

All trials were included for analysis unless the trial failed to capture the walking motion, mostly due to the problem in the human-following algorithm of the robot or when the subjects were too close to the camera; two T10 trials, one T04 trial, and three OW trials were discarded. For the static trial, three seconds of the recording were used in the analysis whereas 20 s of the walking were extracted for the treadmill trials.

For each frame of the recordings used, the poses output by the proposed method is compared with the ones measured by the motion capture system. The motion capture viewed the feet in Marker Frame $F_{marker}$ whose origin was at the IR marker placed at the heel while the RGB-D camera viewed the shoes in Model Frame $F_{model}$. A transformation between the two coordinate systems was necessary to compare the poses. Hence, the first $n_{calib}$ frames of each recording, in which the feet were assumed to be static and the tracker had successfully determined the poses of the feet without error, were used to find the transformation. The average translation was found by computing the arithmetic mean of the translations in the $n_{calib}$ frames. For the rotation, the orientations were first expressed in a $4\times n_{calib}$ matrix of quaternions $Q=[{\vec{q}}_{1}\;{\vec{q}}_{2}\;\cdots \;{\vec{q}}_{n_{calib}}]$. The average quaternion is given to be the normalized eigenvector corresponds to the largest eigenvalue of $QQ^{⊤}$. This transformation was defined to be the transformation between $F_{marker}$ and $F_{model}$; all the poses output by the foot tracker were transformed using the relation to compare the results in the $F_{marker}$.

Two pose errors, in rotation and translation, were generated for each foot. The rotational error is defined as the minimum angle between the orientations measured by the motion capture and the proposed system while the translational error is the minimum distance between the two measurements from the two systems.

As the motion capture system only provides spatial information of the markers, a third-party software, Visual3D v6 Professional by C-motion [35], was used to provide ground truth gait information for validation. The timings of the gait events were labeled manually and the software was used to generate the spatio-temporal parameters of the gait for each step. The HS events detected by the proposed system were examined; between two consecutive HSs, a gait cycle was considered to be detected if there exists a TO event detected in between the two HSs. In the case where multiple TOs were found, the one that was closest to the ideal stance-to-cycle ratio of 60% [36] was used. The gait cycles detected by the proposed system and Visual3D were compared to determine the precision, recall, and F1 score of the step detection:

(19) $$\mathrm{Precision}=\frac{TruePositiveCount}{TruePositiveCount+FalsePositiveCount}$$

(20) $$\mathrm{Recall}=\frac{TruePositiveCount}{TruePositiveCount+FalseNegativeCount}$$

(21) F1=0.5(Precision+Recall).

For each successful cycles detected, the corresponding step length, step width, cycle time, stance time, and swing time were compared to calculate the gait parameter errors. The time discrepancies in the gait events detection were also analyzed.

## 3. Results

**Figure 7:**
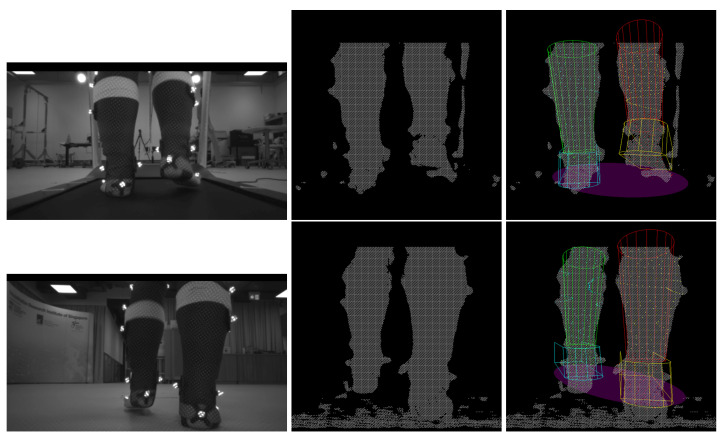
**Top row**: Treadmill trial. **Bottom Row**: Overground trial. **Left column:** Snapshots of the IR image recorded by the RGB-D camera (not used in tracking). **Middle column:** Snapshot of the point clouds. **Right column:** Fitting of the Foot-Shank Models onto the scene. Green and red models represent the left and right shanks, respectively. Cyan and yellow models represent the left and right feet, respectively. Colored points represent the points sampled to fit the models. The purple ellipse represents the subject’s base of support, which can be computed from the foot positions.

Snapshots of the treadmill and overground recordings with tracking results are shown in Figure 7. It can be seen that although the lower limb shapes of the subjects were measured to construct the Foot-Shank Model, the model is still an approximation of the lower limbs. Notwithstanding, despite the simple geometry, the system can still track a subject’s lower limbs movement. The readers are referred to [37] for the videos of the fitting results.

### 3.1. Pose Errors

The pose errors are analyzed in two scenarios, static and dynamic. The static case is found using the static recording while the dynamic pose errors are computed from both the treadmill and overground walking trials. The former identifies the raw fitting accuracy of the models without the influence of the foot movement and the latter mainly establishes the motion tracking performance. Other than individual pose errors for each foot, the relative pose error is also computed; the relative pose is defined as the pose difference between the left and right feet. Relative errors are expected to be related to the spatial gait parameters, which are defined using the pose difference between the feet. These errors demonstrate the effectiveness of the proposed algorithm to be implemented for gait and balance evaluation. The results are tabulated in Table 1.

The static pose errors for rotational and translational errors are less than $13^{\circ }$ and 14 mm, respectively. It shows the raw quality of the model fitting to the observation point cloud without the influence of motion tracking. For the walking trials, T04 has the lowest errors which are less than $20^{\circ }$ and 35 mm for rotational and translational errors, respectively. T10 contributes to the highest errors, which reach as high as $30^{\circ }$ and 70 mm, as well as the highest standard deviations. OW and T10 have comparable errors, albeit the ones of T10 are slightly higher. The walking speed during OW was found to be 0.78±0.11 m/s. The relative errors are smaller than the individual gait errors for all cases except for static translation errors.

### 3.2. Gait Errors

The gait detection performance and gait parameter measurements accuracy determine the effectiveness of the proposed algorithm in gait evaluation. The detection rate of each step is presented in Table 2. The precision of the treadmill trials is more superior than the overground trials, suggesting that they are less susceptible to false alarms. The reader may note that the recall for T04 is low due to a high number of undetected gait, which leads to the lowest F1 score at 0.968. Nonetheless, the F1 scores for all trial types are still greater than 95%.

For all the detected gait cycles, the errors in HS and TO detection times are determined (Table 3). Moreover, five gait parameters, namely step length, step width, cycle time, stance time, and swing time are also compared between the proposed algorithm and the ground truth. The absolute difference are tabulated in Table 4.

Counter-intuitively, the HS error is the highest for T04 and is the lowest for T10. On the other hand, the TO detection errors are comparable among the walking trials. For the temporal gait parameters, T04 contributes to the largest cycle time error at 37 ms; the stance and swing time errors are approximately 70 ms regardless of walking trial type. T04 performs the best in spatial parameters whereas OW is the worst performer, with errors as high as 60 mm and 53 mm for step length and step width, respectively.

### 3.3. Runtime

The tracking system was run on a single CPU thread on Intel Core i7-7567U @ 3.50 GHz using C++ codes. The average computation time for all trials is 42.10 ms per frame (23.75 fps). The times to perform object pixel identification and cost optimization are approximately the same at 20.86 ms and 21.24 ms, respectively. It was found that the identification run time heavily depends on the number of pixels in the image. Hence, if the image is subsampled or has its resolution reduced, it may be able to speed up the process without compromising too much the tracking quality.

## 4. Discussion

This subsection is structured into three parts, namely the quantitative discussion that explains the statistics obtained in the previous section, the qualitative discussion that remarks on the observation from the fitting results, as well as the limitations of the study.

### 4.1. Quantitative Discussion

**Figure 8:**
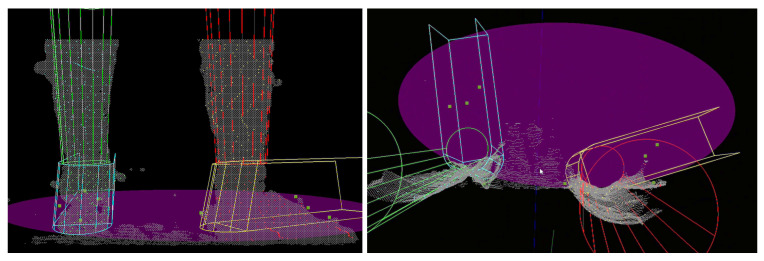
The back (**Left**) and the top view (**Right**) of the starting pose in each trial, which is used to compute the transformation between $F_{marker}$ and $F_{model}$. While it is assumed that the models fit the observation correctly, it often experiences some misalignment as the model shape does not conform to the actual shape of the foot, resulting in an error in the transformation. The dark green points indicate the position of the IR markers, which are placed on the metatarsal heads.

To have a complete view of both the back and the side of the feet, the subjects were required to start each recording with the pose shown in Figure 8. This is when the transformation between $F_{marker}$ and $F_{model}$ is found. However, as seen from the figure, the models, especially the right one, do not align properly to the observation as the side of the foot is not exactly a rectangular plane as described by the model. Hence, the transformation obtained in this configuration carries a degree of discrepancy and thus affects the comparison between the motion capture and proposed algorithm. Despite the discrepancy, the difference in starting poses between both feet does not pose a significant difference to the tracking results as seen in Table 1. To obtain a more accurate fitting, a different model that follows the actual shape of the foot may be necessary. This may require more shape parameters to describe the model. Nonetheless, the side view of the foot is rarely seen unless the subject is performing a turn, which may not be the most common scenario in the application.

The static fitting errors are found to be less than $13^{\circ }$ and 14 mm for rotational and translational errors, respectively. The static pose errors may be caused by a few factors, such as modeling error, sensor noise, IR markers compromising the shape of the lower limbs, time synchronization error between the two camera systems, and the transformation error between $F_{marker}$ and $F_{model}$. Nevertheless, as the average human foot length is 263 mm and 238 mm for the male and female, respectively [38,39], resulting in less than 6% error, the discrepancies are perceived to be acceptable.

During the walking trials, the pose errors increase with the walking speed as the tracker is unable to follow the fast movement of the lower limbs. Nonetheless, it is assumed that the algorithm is still relevant to the intended application of rehabilitation and assistive technologies, in which the users walk slowly [29,30,31,32].

As seen from Table 1, the relative pose errors are smaller than the individual pose errors. This suggests that there exists some constant systematic errors in the individual fitting results, which are canceled out when computing the relative pose. The systematic error is hypothesized to be related to the transformation between $F_{marker}$ and $F_{model}$, as well as the synchronization between the two systems.

To show the effectiveness of the Foot-Shank model, a new method that computes the foot position directly from the point cloud clusters is examined. The object recognition of the new method is the same as the proposed algorithm except that no foot/shank separation is performed; the center of the grouped cluster is directly output as the foot position. The results are tabulated in Table 5. The tracking errors are much larger than the case when the Foot-Shank Model is adopted (58 mm translation error compared with 30 mm for T04 trials). This shows that modeling the lower limbs helps to improve the accuracy and provides the foot orientations, which is necessary to compute the BoS accurately.

The large number of false negatives in gait detection in T04 is caused by undetected TOs. This is prominent in the low-speed treadmill cases as the stance foot moves along with the treadmill belt. The low speed causes the algorithm to be unable to identify the local minimum in the AP-distance of the foot because of the gradual changes, causing low recall and F1 score in T04. This shows that false negatives in low gait speed may be one of the limitations of the proposed algorithm, which may pose a problem in tracking gait impaired subjects.

The errors in gait parameters are significantly affected by the gait events detection of the system. As seen in Table 3, the error can be as high as 76 ms. It directly impacts the accuracy of the cycle time, stance time, and swing time. In addition, the lower limbs may have been displaced significantly during this period, changing the relative position between the feet greatly and hence resulting in a different step length and step width. Among the gait parameters, step length is one of the major indicators for patient recovery. After physical therapy intervention for four to eight weeks, stroke patients can experience a change in step length ranging from 3 cm to 19 cm [40,41], while the change is 6 cm on average [42]. Since the patients walk at 0.4–0.5 m/s [42], the proposed algorithm can still identify the parameter change for most patients.

The errors in gait parameters are normalized by their ground truth gait parameters, i.e., $\%error=\frac{parameter_{measured}-parameter_{true}}{parameter_{true}}\times 100\%$, to have a better understanding of their significance (Table 6). The errors in temporal parameters are 3%, 10%, and 14% for cycle time, stance time, and swing time, respectively. While the spatial parameters vastly differ from each other, the step length percentage error falls around 12%; the step width percentage error is higher as the step width is much shorter than the step length.

One may question why the gait parameter accuracy measured in OW is poorer than T10 despite having a better pose tracking accuracy. During OW, some subjects were seen to adopt unnatural gait behavior of near-tandem walking, presumably affected by the human-robot interaction. This often causes the swinging leg to be obstructed by the lagging leg at the moment of HS. The current algorithm has limitations in handling such obstruction, leading to a larger tracking error at such instances. When the pose of the foot around the instance of HS cannot be determined accurately, the accuracy of the HS timing and the gait parameters computed at the instance are thus impacted. The near-tandem walking also resulted in a very small step width (<30 mm), yielding a large step width percentage error in the case of OW (>126%).

In Visual3D, the relative distance of the feet is computed between the “proximal end positions of the feet”, which is the point of connection between the Visual3D shank and foot model. This may be different from the proposed algorithm in which the distance is computed according to the origin of the Foot-Shank Model. The difference in the definitions may be one of the factors of the spatial gait parameter errors.

### 4.2. Qualitative Discussion

**Figure 9:**
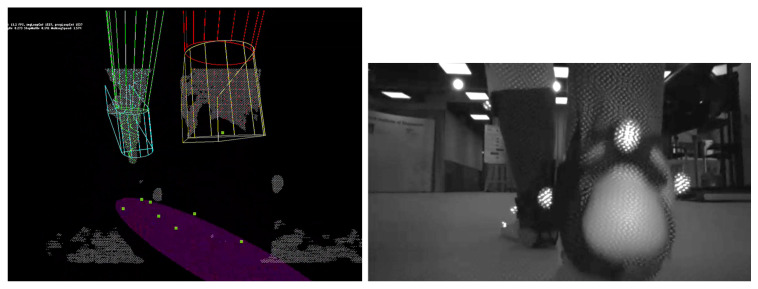
The right foot moves too close to the camera, causing it unable to be seen while blocking the left foot from the view. **Left**: Point cloud with inaccurately fitted model, **Right**: IR image.

There are instances where the foot was so close to the camera that it moved out of its sensing range, making it unseen while blocking the other foot, as shown in Figure 9. This is commonly found during high gait speed or during OW in which the subject walked in a tandem fashion. A camera with a closer sensing range may be necessary to mitigate the problem.

**Figure 10:**
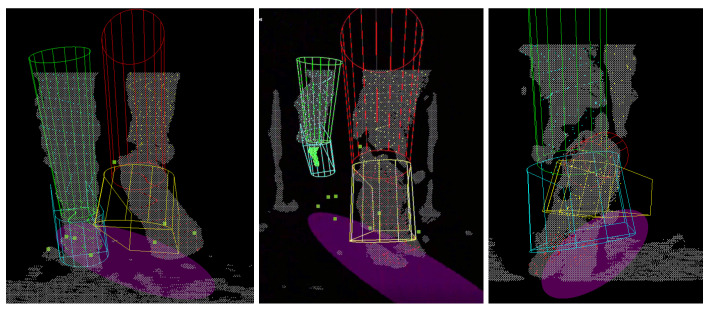
**Left:** The model is unable to follow the observation point cloud during toe-off when the tilting angle of the foot is too large. **Center:** The right foot occludes the left foot which is farther away from the camera. **Right:** As the object gets closer to the camera, it may also fracture into pieces, posing difficulty to the identification algorithm. In this case, the left foot is incorrectly assigned to the right limb, pulling the right model to the left foot.

During the toe-off, the foot was often observed to be in plantar-flexion. The algorithm was unable to conform to this pose when the tilting angle was too large, causing the orientation of the model to be inaccurate (Figure 10, Left). This was caused by two reasons. Firstly, in the current algorithm, the Foot-Shank Separation Line starts at the bottom of the point cloud cluster and advances upwards as the optimization cost decreases. If the optimization cost increases with the new line, the process stops, and the previous line is determined as the actual Separation Line. When the foot is pointing down during toe-off, the separation line is often located at a higher position in the image. Hence, it is unlikely for the output cost to decrease monotonously throughout the iterations. One alternative is to perform fitting with multiple separation lines, which is too time-consuming for the application. The second reason for the misfit is the shank orientation cost imposed in Equation (12), which prevents the model from following the tilt of the shank. However, the cost function is necessary to maintain the shank in an upright position; the model was seen to flip over when the term was disabled.

Occlusion between the legs was also common in normal walking even when the feet were not in camera proximity. While the tracking could be suspended if the other limb was completely obstructed, this was not commonly the case; in most scenarios part of the limb was still observable, causing erroneous results as the algorithm was unable to detect which part of the lower limb was occluded. As shown in (Figure 10, Center), the right foot obstructed the left foot in front, leaving only the shank to be visible. The left model was then “forced” to move up to align to the sampled points. The system should be improved to handle such situations more robustly by recognizing which part of the lower limb is present in the image.

Moreover, as the object moves closer to the camera, the image may fracture into multiple pieces, posing an issue to the current algorithm as the system is unable to correctly cluster the fracture parts to the correct limb (Figure 10, Right). This poses an issue in the identification algorithm which identifies the left and right limbs by sorting clusters of point clouds into two groups. In some cases, the fractured clusters may be sorted into the wrong limb, forcing the model to fit to the points which are on the other lower limb. A more robust object identification algorithm is necessary to overcome the problem, such as using machine learning in image recognition. Notwithstanding, albeit having high accuracy, such a machine learning algorithm often requires the use of GPU, which increases the cost of the assistive devices.

### 4.3. Limitations

**Figure 11:**
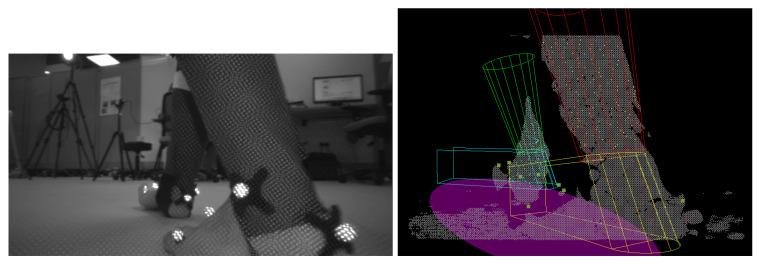
During turning, the foot may move to the opposite side of the image. The current method is unable to cover this scenario and will hence wrongly assign the right model (red and yellow model) to the left foot and vice versa.

Although turning motion is not studied in this paper, the condition was recorded during the experiment. In the current algorithm, the left and right limbs are determined by their relative positions in the depth image. While this may be appropriate in the case of treadmill use, it poses a problem in overground turning motion in which the feet swap positions, as shown in Figure 11. An alternative method is necessary to localize the lower limbs. A simple idea is to infer the moving direction of the subject from the motion of the robot. In the case of turning, the feet may be tracked with the help of their previous positions; otherwise, the feet are assumed to be located at their expected positions in the image.

The runtime of the object identification and optimization requires more than 40 ms, which is slower than the update frequency of the RGB-D camera. Although the runtime can be reduced by subsampling the image into a lower resolution, the program may still not be fast enough to capture fast motion in case of higher gait speeds. Nonetheless, this may not be a critical problem in the application as the system is designed for gait impaired subjects who walk slowly.

The algorithm has only been tested with healthy young subjects with no known locomotion disability. While slow-walking generally yields better results (<$20^{\circ }$ and 35 mm fitting error for rotation and translation, respectively), it is unknown if the performance will be the same for patients with pathological gaits. The error in gait parameters (as high as 30% for step width) shows that it is significantly affected by the accuracy of HS and TO detection timing. The slow motion of patients may pose an issue to the current gait events detection algorithm, as seen in the large number of false negatives in T04.

The algorithm assumes that the only objects in the scene are the lower limbs of the user. While tracking will be affected by walking aids, such as walkers and canes, such equipment may not be necessary when the assistive robot is maintaining the balance of the user. Nevertheless, the system may need to be improved to handle scenarios in which the user is wearing long loose pants that will compromise the rigid object tracking.

## 5. Conclusions

In summary, this paper presented a geometric model of the lower limb that could be applied in foot tracking when viewing the lower limbs from the back using a depth camera. It aligns the model to the point cloud scene using optimization to localize the feet. The proposed algorithm provided a contactless, portable, and cost-effective method running on CPU only, without requiring the user to wear any customized footwear, giving it the potential to be implemented into overground rehabilitation and assistive robots to evaluate the performance of gait-impaired individuals. The tracking errors during slow walking (0.4 m/s) that are commonly found in gait impaired individuals were found to be <$20^{\circ }$ and 35mm for rotation and translation, respectively. Gait parameters were also extracted from the fitting results, which had errors of <35 mm for step length and step width, and <38 ms for cycle time. The errors for stance time and swing time were slightly higher at around 70 ms.

The fitting results could also be used to construct the Base of Support (BoS) of the subject for balance evaluation. To do this, the key model points the form that foot models are projected on the ground plane. The projected points are then used to fit an ellipse, which is assumed to be to the BoS of the subject. It can be seen from the images (e.g., Figure 7) that the results look realistic when the model fitting is accurate. Nonetheless, this has to be validated with the motion capture system and force plates.

The next step is to address the limitations discussed, such as improving the robustness of the fitting and the runtime of the algorithm. The system will also be evaluated on pathological gaits to show its feasibility in the intended application.

## Figures and Tables

**Table 1:** The pose errors for both feet and the relative pose error.

Experimental Conditions	Left		Right		Relative ^1^	
	Rotation (°)	Translation (mm)	Rotation (°)	Translation (mm)	Rotation (°)	Translation (mm)
Static	11.35 ± 9.01	11.31 ± 6.98	12.45 ± 6.30	13.79 ± 4.41	5.31 ± 3.91	11.32 ± 9.24
Overground	19.69 ± 13.94	63.16 ± 61.93	26.71 ± 15.89	62.25 ± 52.65	9.16 ± 7.52	26.38 ± 35.79
Treadmill 0.4 m/s	19.08 ± 7.61	29.51 ± 19.94	17.86 ± 9.07	33.09 ± 27.26	6.84 ± 4.86	14.59 ± 11.61
Treadmill 1.0 m/s	24.95 ± 17.91	66.09 ± 63.16	28.84 ± 22.49	70.59 ± 62.20	10.51 ± 8.85	28.73 ± 30.95

^1^ Relative is defined as the pose difference between the left and right feet.

**Table 2:** The detection statistics of the steps for different types of walking trials.

Experimental Conditions	TP ^1^	FP ^2^	FN ^3^	Precision ^4^	Recall ^5^	F1 ^6^
Overground	283	13	4	0.956	0.986	0.971
Treadmill 0.4 m/s	456	2	28	0.996	0.942	0.968
Treadmill 1.0 m/s	705	8	0	0.989	1.000	0.994

^1^ True Positive Count. ^2^ False Positive Count. ^3^ False Negative Count. ^4^ $\frac{\mathrm{TP}}{\mathrm{TP}+\mathrm{FP}}$. ^5^ $\frac{\mathrm{TP}}{\mathrm{TP}+\mathrm{FN}}$. ^6^ 0.5 × (Precision + Recall).

**Table 3:** The heel-strike and toe-off detection errors.

Experimental Conditions	Heel-Strike (ms)	Toe-Off (ms)
Overground	62.70 ± 51.89	31.15 ± 44.01
Treadmill 0.4 m/s	76.47 ± 37.46	33.98 ± 75.69
Treadmill 1.0 m/s	43.15 ± 25.52	30.71 ± 24.25

**Table 4:** The gait parameter errors for different types of walking trials.

Experimental Conditions	Step Length (mm)	Step Width (mm)	Cycle Time (ms)	Stance Time (ms)	Swing Time (ms)
Overground	60.67 ± 41.63	53.66 ± 38.78	35.12 ± 29.13	69.78 ± 38.63	69.98 ± 42.19
Treadmill 0.4 m/s	34.82 ± 27.67	32.54 ± 20.75	37.72 ± 79.78	72.23 ± 87.75	67.81 ± 46.20
Treadmill 1.0 m/s	53.76 ± 44.80	34.19 ± 24.28	30.26 ± 26.31	66.98 ± 36.05	66.36 ± 36.55

**Table 5:** The pose errors for both feet as well as their relative pose errors when computing the foot positions directly from the point cloud clusters.

Experimental Conditions	Left (mm)	Right (mm)	Relative (mm)
Overground	78.30 ± 45.31	92.58 ± 46.68	76.86 ± 47.48
Treadmill 0.4 m/s	58.54 ± 31.66	90.02 ± 37.25	67.86 ± 48.45
Treadmill 1.0 m/s	83.54 ± 58.08	105.34 ± 53.40	70.32 ± 48.81

**Table 6:** The gait parameter percentage errors for different types of walking trials. $\%error=\frac{parameter_{measured}-parameter_{true}}{parameter_{true}}\times 100\%$.

Experimental Conditions	Step Length	Step Width	Cycle Time	Stance Time	Swing Time
Overground	13.86 ± 9.16	126.48 ± 308.94	3.16 ± 2.54	10.50 ± 5.66	15.40 ± 8.63
Treadmill 0.4 m/s	12.14 ± 10.07	29.02 ± 29.80	2.51 ± 5.49	7.36 ± 9.09	12.60 ± 7.66
Treadmill 1.0 m/s	10.39 ± 8.08	30.71 ± 31.24	2.97 ± 2.52	11.98 ± 6.72	14.71 ± 7.78

## Data Availability

The data generated from the study can be found in https://entuedu-my.sharepoint.com/:f:/g/personal/foom0009_e_ntu_edu_sg/EhbNcsAHcbFEpl63tPB3jcUBriaoLu-_ydxHOZmZifg3Bg?e=cDEgGk.

## Abbreviations

The following abbreviations are used in this manuscript:

AP	Anterior-posterior
BoS	Base of support
HS	Heel strike
IR	Infrared
LM	Levenberg–Marquardt
MRBA	Mobile Robotic Balance Assistant
OW	Overground walking
PCA	Principal component analysis
RGB-D	Red green blue depth
SVM	Support vector machine
T04	Treadmill walking at 0.4 m/s
T10	Treadmill walking at 1.0 m/s
TO	Toe off
XCoM	Extrapolated center of mass
